# Genetic variation for parental effects on the propensity to gregarise in *Locusta migratoria*

**DOI:** 10.1186/1471-2148-8-37

**Published:** 2008-02-01

**Authors:** Marie-Pierre Chapuis, Arnaud Estoup, Arnaud Augé-Sabatier, Antoine Foucart, Michel Lecoq, Yannis Michalakis

**Affiliations:** 1 Centre de Biologie et de Gestion des Populations, Institut National de la Recherche Agronomique, Campus International de Baillarguet, 34988 Montferrier/Lez, France; 2 Génétique et Evolution des Maladies Infectieuses, UMR 2724 Centre National de la Recherche Scientifique – Institut de la Recherche et du Développement, Institut de la Recherche et du Développement, 911 avenue Agropolis, 34394 Montpellier Cedex 5, France; 3 Ecologie et Maîtrise des Populations d'Acridiens, Département BIOS, Centre de coopération internationale en recherche agronomique pour le développement, Campus International de Baillarguet, 34398 Montpellier Cedex 5, France

## Abstract

**Background:**

Environmental parental effects can have important ecological and evolutionary consequences, yet little is known about genetic variation among populations in the plastic responses of offspring phenotypes to parental environmental conditions. This type of variation may lead to rapid phenotypic divergence among populations and facilitate speciation. With respect to density-dependent phenotypic plasticity, locust species (Orthoptera: family Acrididae), exhibit spectacular developmental and behavioural shifts in response to population density, called phase change. Given the significance of phase change in locust outbreaks and control, its triggering processes have been widely investigated. Whereas crowding within the lifetime of both offspring and parents has emerged as a primary causal factor of phase change, less is known about intraspecific genetic variation in the expression of phase change, and in particular in response to the parental environment. We conducted a laboratory experiment that explicitly controlled for the environmental effects of parental rearing density. This design enabled us to compare the parental effects on offspring expression of phase-related traits between two naturally-occurring, genetically distinct populations of *Locusta migratoria *that differed in their historical patterns of high population density outbreak events.

**Results:**

We found that locusts from a historically outbreaking population of *L. migratoria *expressed parentally-inherited density-dependent phase changes to a greater degree than those from a historically non-outbreaking population.

**Conclusion:**

Because locusts from both populations were raised in a common environment during our experiment, a genetically-based process must be responsible for the observed variation in the propensity to express phase change. This result emphasizes the importance of genetic factors in the expression of phase traits and calls for further investigations on density-dependent parental effects in locust phase change. More population samples with different outbreak histories need to be analyzed to demonstrate that differences in propensity to gregarise evolve because of different outbreak histories.

## Background

Phenotypic plasticity, the capacity of a genotype to produce different phenotypes in different environments, is ubiquitous in nature [1]. It is now clear in many cases that phenotypic plasticity is adaptive, allowing organisms to exploit temporally and spatially heterogeneous environments [2]. When the environmental cues that mediate phenotypic change vary both geographically and temporally, the potential exists for the evolution of population-level differentiation in the expression of phenotypic plasticity [3-5].

Non-genetic parental effects deserve particular attention in light of current theoretical interest in the evolution of plasticity [6]. These transgenerational effects can be viewed as the plastic phenotypic response of the offspring to parental environmental conditions [7]. Although parental effects on reaction norms for morphological, behavioural, and physiological traits have been investigated in a variety of organisms [reviewed in 8 for insects], only recently have environmental parental effects been viewed as a form of plasticity that may itself evolve [7,9]. If environmental parental effects are important in natural populations, genetic differentiation among populations in reaction norms induced by parental environments may be fairly common, as is the case for the differential expression of other types of phenotypic plasticity [10]. Unfortunately, little is known about the extent of such genetic variation for parental effects on offspring phenotype among populations occupying different habitats and experiencing different levels of selection. This is especially true for animals (for plants see [7,10]).

Many insect species exhibit plastic changes in physiology, behaviour and morphology in response to crowding [11]. Crowding-dependent phenotypic plasticity is expressed to varying degrees among members of the Coleoptera, Lepidoptera, Hemiptera, Homoptera, and Orthoptera [11]. However, the ability to gregarise, in which individuals undergo extreme phase change from the low population density solitarious phase to the high population density gregarious phase, is a defining feature of locusts [12-14]. This remarkable phase change has been interpreted as an adaptation for migration [15] at high population density that reduces high risks of predation [16], disease [17], or competition for food and mates [18,19]. Environmental parameters have rapidly emerged as primary causal factors of phase change in locusts [20]. Phase change depends primarily on population density within the lifetime of both offspring and parents [13,21,22]. The changes induced by the high density environment of the parent drive a positive feedback loop that promotes additional gregarisation across generations. Thus, parental effects are central to the extreme nature of the density-dependent phase change in locusts, as well as the time-course of its expression in the field. What remains unclear, however, is the potential role of genotype-dependent variation in the expression of phase change, and in particular, variation in response to the parental environment.

*L*. *migratoria *presents a unique system in which to examine genetic variation in the expression of locust phase change because geographic variation exists among populations in their propensity to outbreak and form swarms [23]. Swarming and the expression of gregarisation are highly correlated among *L. migratoria *populations [24]. Furthermore, patterns of geographic variation among populations in the propensity to gregarise reflect underlying patterns of temporal variation in locust population densities. Typically, *L. migratoria *individuals are found at low densities in the solitarious phase. In historically outbreaking areas, the species displays huge increases in local population densities at irregular intervals. These high density conditions generate swarming individuals typical of the gregarious phase.

In this paper we report the results of an experiment investigating the reaction norms of two commonly studied sets of phase-related traits, morphometry of adults and behaviour of nymphs. We compared the phenotypic responses to parental density conditions of locusts from two naturally-occurring, closely-related populations of *L. migratoria *with contrasting historical patterns of outbreak events, namely a frequently outbreaking population from Madagascar and a non-outbreaking population from France. We first reared both populations under isolated conditions for two generations to control for unknown parental histories in the field, then reared both populations under isolated or crowded conditions for two generations, and finally quantified phase traits of progeny all of which were under homogeneous isolated conditions. Because all assayed individuals were raised in the same environment, our design eliminated proximal environmental effects and allowed us to ascribe potential differences to population-level divergence in the effects of parental density on the expression of progeny phase traits. We found that locusts from a historically outbreaking population of *L. migratoria *expressed parentally-inherited phase change to a greater extent than locusts from a historically non-outbreaking population, providing new insight into the evolution of phase change.

## Results

**Figure 1:**
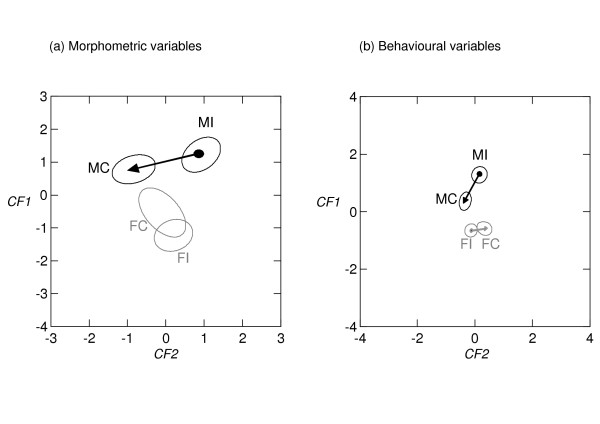
**First two canonical functions for the four groups of locusts obtained by canonical discriminant analysis of the measurements of four morphometric variables (a) and eleven behavioural variables (b)**. Note: *MI*, Locusts from Madagascar with an experimental rearing history of isolation; *MC*, Locusts from Madagascar with an experimental rearing history of crowding; *FI*, Locusts from France with an experimental rearing history of isolation; *FC*, Locusts from France with an experimental rearing history of crowding; *CF1*, First canonical function; *CF2*, Second canonical function. The ellipses correspond to 95% confidence ellipses. Arrows show the significant distances between the centroids of groups from the same population of origin with experimental rearing histories of isolation and crowding, as well as directions of gregarisation.

Before proceeding to the analysis of the interactive effects between POPULATION and HISTORY, we first briefly report the "baseline" differences in morphometry and behaviour between the French and Malagasy populations (POPULATION effects in the full factorial MANOVAs with Box-Cox transformed data; *P *< 0.0001). French locusts had eyes spaced farther apart, a smaller ratio of the length of the fore wing to the length of the hind femur, and a larger ratio of the length of the hind femur to the maximum width of the head than Malagasy locusts (first canonical function *CF1 *of the canonical discriminant analysis of the morphometric data; see Figure 1a and Additional file 1). In their behaviour, French locusts had a more tortuous track, spent less time climbing, had a lower track speed, spent more time walking, and jumped more frequently than Malagasy locusts (*CF1 *of the canonical discriminant analysis of the behavioural data; see Figure 1b and Additional file 1). Such inter-population differences in the solitarious phenotypes of *L. migratoria *have already been reported by previous studies [25] and are expected through local adaptation to the French temperate vs. Malagasy inter-tropical habitats.

Rearing history affected the expression of phase traits in locusts from the Malagasy and French populations in different ways. This differential response was apparent in the significant interactive effect of POPULATION and HISTORY on both the morphometric and behavioural phase traits (POPULATION × HISTORY in the full factorial MANOVAs with Box-Cox transformed data; *P *= 0.0008 and *P *< 0.0001, respectively; Table 1). The first two canonical functions, obtained by the canonical discriminant analysis of the measurements of the four morphometric variables, yielded significant differences among group centroids (*CF1*: Wilks' Lambda; *χ*^2^_12 _= 95.6 ; *P *< 0.0001; second canonical function *CF2*: Wilks' Lambda; *χ*^2^_6 _= 32.3; *P *< 0.0001; see Additional file 1 for more details on the two canonical functions) and accounted for 98% of the variation between groups (Figure 1a). History did not significantly affect morphometric traits of the French population, in contrast to the Malagasy population (test on Mahalanobis distances: *P *= 0.0825 and *P *< 0.0001 respectively). Malagasy *F*/*C*, *DE*, and *H*/*P *values decreased with crowding history (forward stepwise selection procedure with *F *= 2.706 on Malagasy groups with histories of isolation and crowding), in agreement with previous morphometrical studies [25].

**Table 1:** MANOVAs of the effects of POPULATION, HISTORY and their interaction on the overall morphometry and on the overall behaviour of the four groups of locusts.

Data	Source	*DF*	Wilks' Λ	*F*	*P*
Morphometry	POPULATION	4	0.5182	20.5	< 0.0001
	HISTORY	4	0.7750	6.4	0.0001
	POPULATION × HISTORY	4	0.8090	5.2	0.0008
	Residual	88			

Behaviour	POPULATION	11	0.6435	16.8	< 0.0001
	HISTORY	11	0.9060	3.1	0.0005
	POPULATION × HISTORY	11	0.8740	4.2	< 0.0001
	Residual	331			

The four MANOVA test statistics Wilks' Λ, Pillai's trace, Hotelling-Lawley trace and Roy's maximum root concluded that *H*_0 _is rejected for each source of variation for both morphometric and behavioural data. The results presented in the table are those obtained with Wilks' Λ test statistic.

The first two canonical functions, obtained by canonical discriminant analysis of the measurements of the eleven behavioural variables, yielded significant differences among group centroids (*CF1*: Wilks' Lambda; *χ*^2^_33 _= 208.9 ; *P *< 0.0001; *CF2*: Wilks' Lambda; *χ*^2^_20 _= 49.4; *P *= 0.0003; see Additional file 1 for more details on the two canonical functions) and accounted for 91% of the variation between groups (Figure 1b). The French population expressed a substantially less pronounced behavioural gregarisation relative to the Malagasy population (test on Mahalanobis distances: *P *= 0.0122 and *P *< 0.0001 respectively). Behavioural changes induced by crowding history in the Malagasy population were typified by an increase in activity, consistent with previous analyses of locust behaviour [26]. Malagasy locusts exhibited density history-dependent increase in turn frequency and decreases in climb time and swaying frequency. On the other hand, the differences among the French locusts were relatively minor; those with a crowded history increased their swaying frequency and decreased their track speed (forward stepwise selection procedures with *F *= 2.706 on groups with histories of isolation and crowding from Madagascar and France, respectively).

Altogether, these results indicate that locusts from a historically outbreaking population from Madagascar express a more pronounced phase change when subjected to a crowded-history than do locusts from a historically non-outbreaking population from France.

## Discussion

The most comprehensive theory of locust phase change at the moment is that the switch towards gregarisation primarily depends on environmental conditions, in particular population density [20]. In this study, we found that locusts from a historically outbreaking population of *L. migratoria *from Madagascar expressed larger behavioural and morphometric phase changes than those from a related historically non-outbreaking population from France. Because both populations were raised in a common environment during our experiment, a genetically-based process must be responsible for this variation in the propensity to gregarise. Furthermore, our experimental design indicated that this genetic difference between the two populations is in the expression of parentally-inherited gregarisation. It is worth noting that our experimental procedure was conservative in revealing such parental effects because measured individuals of both phases were reared in identical isolation conditions, which should only decrease the differences between the treatment groups. The existence of genetic variation in the reaction norm for a phenotypic response to parental environments further underlines the importance of environmental parental effects in the evolution of plasticity in natural populations. The physiological factors underlying the population-level variation in the expression of parental effects remain to be determined. The parental effects involved in the change from the solitarious to the gregarious phase have been shown to be transferred in a dose-dependent manner by a water-soluble gregarising factor added to the egg foam in *Schistocerca gregaria *[27]. Assuming a similar physiological mechanism in *L. migratoria*, mothers of the non-outbreaking population may have secreted smaller quantities of, or a less effective form of the factor than mothers of the outbreaking population. Alternatively, offspring of the non-outbreaking population may have been less sensitive to the gregarising factor than offspring of the outbreaking population.

The genetic difference between one outbreaking Malagasy and one non-outbreaking French *L. migratoria *populations suggests that the outbreak history may play a critical role in the evolution of phase change expression. However, more population samples are required to demonstrate the adaptive nature of the observed population differences in relation to the outbreak history. Unfortunately, it proved logistically impossible to obtain large enough samples from more populations of each outbreaking history at the same time. It was important for our experiment to use samples isolated from the field at exactly the same time, such that they have the same rearing history prior to the treatments, precisely because we wanted to test for an effect of rearing history. Generalising our results to the entire *L. migratoria *species must also be done with caution. Further experimental work is hence needed to confirm that non-outbreaking populations may express phase change to a lesser degree than outbreaking populations in *L. migratoria*. However, our results do unambiguously show that genetic differentiation must be responsible for the variation in the propensity to gregarise of at least some populations. This finding incites for further research elucidating the respective roles of proximate environmental versus evolutionary genetic factors in promoting locust phase change.

An intraspecific differential evolution in the ability to gregarise, as suggested by our comparative study of two populations, may play an important role in swarming patterns across the species range of *L. migratoria*. Global changes, primarily land use change [e.g., in Indonesia: [28]] but also warming and drought [e.g., in China: [29]], are progressively creating new favourable environments for the migratory locust in historically non-outbreaking areas. The risk of increasing outbreak events in such areas would be closely related to the genetic ability to gregarise of the populations of interest. This, in turn, depends on the amount of effective gene flow with populations able to change phase and the time since selection for the gregarious phase was relaxed. Outbreaks have not been observed in Sumatra, Borneo, and New Guinea until recently, where serious outbreaks have occurred, correlated to anthropogenic changes favouring increases in population density [28]. Further research would be needed to determine the extent to which these recent outbreaks are due to selection for gregarisation among autochthonous locusts or to immigrant locusts able to change phase.

The differential evolution of the phase change between two closely related populations of *L. migratoria *also provides new insight into the evolutionary diversification within the family Acrididae. The ability to change phase from solitarious to gregarious in response to population density increase has evolved multiple times within this family, resulting in the phylogenetically heterogeneous group of 'locusts' [13]. In particular, the swarming character has evolved three times within the genus *Schistocerca*, with the more frequently swarming species, *S. gregaria*, at the base of the phylogeny [30]. Behavioural propensities to gregarise among frequently and rarely swarming related *Schistocerca *species have been shown to evolve differentially [31]. Moreover, some other grasshopper species have also been shown to express some gregarious phase-like characteristics under crowded conditions, such as melanisation (e.g., *Schistocerca lineata *[32]), aggregation behaviour [33] or increased metabolism (e.g., *Aiolopus thalassinus *[34]). This distribution of the gregarious phase traits across taxa, seemingly independent of the phylogeny of the group, is consistent with the hypothesis that genetic variation for phase change may have been present during the evolutionary past of many Acridids, but that the plasticity for these traits has been lost many times via genetic assimilation [35,36]. Through this process, reaction norm evolution can transform initially plastic phenotypes into genetically determined constitutive phenotypes [37]. Our demonstration that a *L. migratoria *population lacks, at least partly, the genetic capacity to mount the appropriate density-dependent responses, lends further support to the evolution of locust phase change via genetic assimilation. The process of genetic assimilation has been invoked to explain many instances of phenotypic evolution, and in particular the evolution of warning coloration in grasshoppers, a trait that can be density-mediated [35].

One important methodological issue arises from our results. To date, phase change has usually been analysed by directly comparing individuals reared in isolation to individuals reared under crowded conditions. In particular, this approach was used in two previous studies examining intraspecific genetic variation in locust phase change. Results of these experimental studies were unclear and conflicting. In the non-outbreaking species *Schistocerca americana*, first-instar nymphs exhibited geographic variation in their expression of changes in behaviour with crowding, but fifth-instar nymphs did not [31]. On the other hand, *in L. migratoria*, Heifetz et al. [38] found that the morphometry of locusts from a historically non-outbreaking population was not affected by crowding, but that they were unexpectedly more active than locusts from a historically outbreaking population. Importantly, phase change is distinct from many other examples of phenotypic plasticity in that the environment of the parents can also affect the phenotypes of their offspring [21]. Thus, the common approach of directly comparing isolated and crowd reared locusts potentially confounds the proximal density effects, resulting from an individual's own experience, with environmentally inherited (e.g., non-genetic parental) effects. This confusion may at least partially explain the contradictory results of previous studies. Our study highlights the importance of studying phase change inherited by isolation or crowding histories (i.e., phase change that accrues across generations) instead of that expressed in response to isolated or crowded conditions experienced within the lifetime of an individual. Our experimental design allowed us to cast off the confounding density effects, despite the fact that appropriate experimental protocols are time and manpower demanding (five generations of rearing vs. one typically). Proximal density effects may also confound the results of whole-genome expression analyses of locusts that have just come within reach in the effort to identify the major genetic mechanisms underlying phase transition [39]. Kang et al. [40] identified more than 500 differentially expressed genes between laboratory induced solitarious isolated and gregarious crowded *L. migratoria *individuals. This result may suggest that molecular bases of phase change are elaborate. However, many of the gene expression differences may actually not be related to phase differences but to the different laboratory environments in which the two phases were reared. Hence, our study suggests that a promising approach to investigate the molecular basis of locust phase change would be whole-genome comparisons of locusts expressing phase traits inherited under isolation or crowding histories to cast off the potentially confounding proximal effects of rearing density.

## Conclusion

This study demonstrates genetic variation in the expression of phase change between one outbreaking population of *L. migratoria *from Madagascar and one non-outbreaking population from France. This intraspecific difference highlights the fact that while phase change is environmentally determined, its degree of expression is under genetic control, and can differentially evolve via natural selection (or genetic drift) in different populations. The existence of genetic variation in the reaction norm for a phenotypic response to parental environments further underlines the importance of environmental parental effects in the evolution of plasticity in natural populations. More population samples are needed to prove the role of the outbreak history in the differential expression of phase change among *L. migratoria *populations.

## Methods

### Populations

*L. migratoria *insects for this experiment were collected in two sites, Betioky (South-Western Madagascar) and Narbonne-plage (Southern France). The two populations are characterized by contrasting historical patterns of outbreak events. The intertropical environment in Madagascar is cyclically favourable to increases in population density; five intense outbreaks and two incipient outbreaks controlled by insecticides have been recorded during the last century [41]. The population from Madagascar was thus considered as a historically frequently outbreaking population. In Southern France, only a single outbreak originating from the Western coast of the Black Sea has been reported dating back to the XIV^th ^century [42]. The population from France was therefore considered as a historically non-outbreaking population. At the moment of sampling, field densities were low (i.e., 200 and 280 adults per ha for the Malagasy and French populations respectively; results not shown), well below the critical density for first phase change manifestations (i.e., 2000 adults per ha [43]). All individuals collected from the Malagasy and French populations corresponded to the solitarious form. The locusts from Madagascar were sampled three years after the last outbreak event recorded (i.e., after approximately 12 generations assuming a generation time of four generations per year [43]). The two sampled populations were closely related as shown by genetic analysis at neutral microsatellite loci (*F*_ST _= 0.083; [44] and results not shown).

### Experimental design

**Figure 2:**
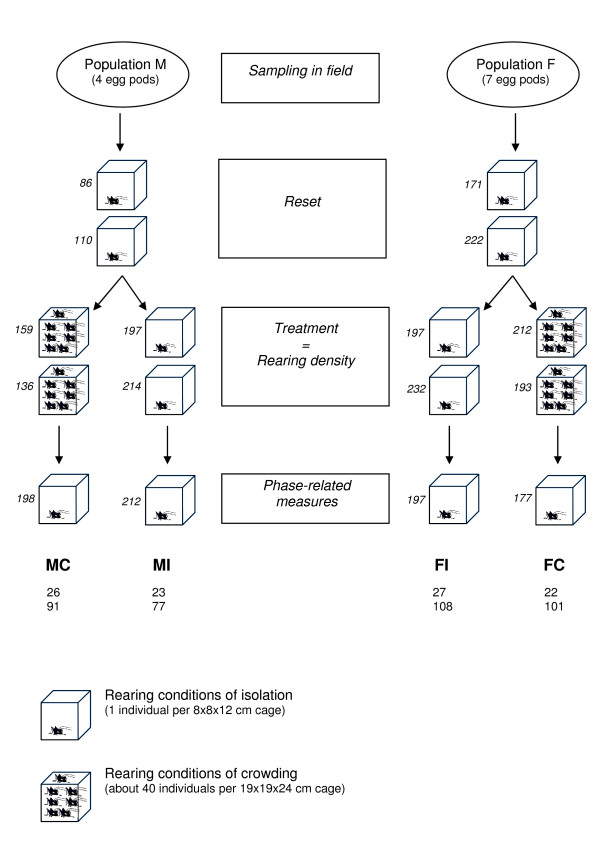
**Experimental design and locust groups obtained**. *M*, Individuals originating from Madagascar; *F*, Individuals originating from France; *MI*, Individuals originating from Madagascar with an experimental rearing history of isolation; *MC*, Individuals originating from Madagascar with an experimental rearing history of crowding; *FI*, Individuals originating from France with an experimental rearing history of isolation; *FC*, Individuals originating from France with an experimental rearing history of crowding; *G*_0_, Generation of sampling; *G*_1 _to *G*_5_, First to fifth generation of experimental rearing. The numbers of larvae used to initiate each generation and treatment are shown in italicized characters. Numbers below each group (*MI*, *MC*, *FI*, and *FC*) indicate the numbers of insects measured for morphometry (first line) and behaviour (second line). See text for more details.

Disentangling the phase change that accrues across generations from that occurring within the lifetime of an individual requires a close control of the density conditions experienced by an individual across generations and homogeneous environmental conditions at the stage of measurements. The population density encountered in the field by the Malagasy and French populations might differ even if we sampled solitarious-like locusts in both cases. In particular, populations of South-Western Madagascar might experience high density conditions during the rainy season, and some gregarious populations were occasionally observed in this area. Since the parental density is known to affect the phase characteristics across generations in locusts [27], a potential source of variation in the expression of gregarious phase traits between two populations may be due to uncontrolled parental effects. To control for parental histories, both populations were reared in isolated conditions for two generations after sampling. In *Schistocerca gregaria*, the behavioural change acquired after short periods of crowding is rapidly lost [45]. Hence, if we assumed a similar time-course of behavioural phase change in *L. migratoria*, the insects were solitarious at the end of this reset step. We then reared individuals under isolated or crowded conditions for two subsequent generations. Finally, we measured phase-related traits of insects in the next generation (i.e., 5^th ^generation) that had been reared in individual cages. Figure 2 presents an overall view of the experimental protocol and the resulting four groups we obtained: Malagasy population with a history of isolation (*MI*), Malagasy population with a history of crowding (*MC*), French population with a history of isolation (*FI*), and French population with a history of crowding (*FC*). To prevent the loss of genetic diversity due to drift, each generation started with 86 to 232 larvae from different egg pods (at least 14). As a result, genetic variation among the individuals within each of the four groups was substantial as measured by the expected heterozygosity (0.53 ≤ *H*_*E *_≤ 0.80) at neutral microsatellite loci ([44] and results not shown).

Because the measured individuals from treatment groups were raised in a common environment, differences between them cannot be ascribed to differences in their environmental conditions, but only to differences in their origin (Madagascar or France) and/or their rearing history. However, phase-related traits of the measured individuals under different treatments also depend on the density conditions these individuals experienced themselves. Adults measured for morphometrics were reared alone throughout their entire lives. Hatchlings measured for behaviour were separated from siblings on the day of emergence, and so only remained together for a few hours after which they were reared in isolation for the rest of their lives. In *Schistocerca gregaria*, nymphs treated by crowding for a few hours and then re-isolated for only one hour were statistically similar in behaviour to untreated nymphs [45]. Assuming a similar time-course of behavioural phase change in nymphs of *L. migratoria*, contact with siblings may have only slightly affected the measured behaviours of newly hatched nymphs (1 day post hatching), and so their designated environment remained "isolation". Consequently, for both Malagasy and French crowding-history groups, parental experience of crowding conditions might be partly counter-balanced by the individual experience of isolation conditions. This counter-balancing effect on measured "gregarisation" makes our experimental design conservative. The fact that the Malagasy population showed a larger propensity to gregarise than the French population in spite of the potential confounding effect of rearing measured individuals in isolation strengthens our conclusions.

### Rearing

The locusts were maintained under either isolated or crowded conditions. Isolated rearing facilities were similar to those described in Hoste et al. [46], with slight modifications. Although the most potent stimulus causing solitarious locusts to assume gregarious traits is physical contact [47], the combination of visual and olfactory stimuli is also gregarising [48]. Consequently, we rendered cages opaque to visually isolate insects from each other. We also homogenized light conditions within each drawer by adding 'cool' tube lamps every two shelves and we ventilated cages through a holed top. Crowd-reared locusts were kept in a separate room in 19 × 19 × 24 cm cages at a density of about 40 individuals per cage. Different cages (at least three) were used for each population at each generation of crowded rearing (i.e., 3^rd ^and 4^th ^generation; Figure 1). Cages were ventilated and lit in a similar way to that of the individual cages. Isolated and crowded locusts were reared under completely identical room and feeding regimes. Rooms were maintained under a 14 h light/10 h dark cycle, a fluctuating 14–10 h temperature regime of 32–28°C, a constant humidity of 50%, and one complete air renewal every 3 min to minimize olfactory contact among cages. Insects were fed every two days with seedling wheat, supplemented by wheat bran for adults. Upon reaching sexual maturity, isolated females and males were placed together for 48 h to ensure insemination. Mating was done between isolated adults originating from different parents by using their reference numbers. For both isolated and crowded conditions, egg pods were obtained in 100 × 50 mm diameter plastic tubes filled with moist sand (10 parts sand; 1 part water). Following oviposition, tubes were monitored for hatching which typically occurred in 12 days.

### Assaying phase state

Because phase change is a composite character, efforts to define phase state based on a single trait that changes during phase transition are illusory [49]. We considered two widely used sets of phase characteristics: morphometry, which is traditionally used in the field, and behaviour, which is widely used since the recent establishment of an assay and descriptive framework for locust behaviour [reviewed in [26]].

Four morphometrical variables were considered (see Additional file 2 for illustrations of the measurements): (i) the ratio of the length of the fore wing on the length of the hind femur; (ii) the ratio of the length of the hind femur on the maximum width of the head; these two ratios are commonly used for characterizing morphometrical phase state [50]; (iii) the ratio of the maximum height of the pronotum on the length of the pronotum, which translates the well-known opposition between the quite convex pronotum of solitarious locusts and the flat pronotum of the gregarious type; (iv) the minimum distance between the eyes, which seems to be one of the most discriminating phase variables [51]. We measured only adult females (40 days post-hatch) to cast off the sex-specificity of morphometrical phase change [25]. For each group (*MI*, *MC*, *FI*, *FC*), 22 to 27 insects were measured with electronic sliding callipers (accuracy 0.01 mm).

We also employed an individual-based behavioural assay developed by Roessingh et al. [52] with slight modifications. We observed 77 to 108 first-instar nymphs (1 day post-hatching) per group. Each locust was introduced via a modified syringe into the middle of a rectangular arena (30.5 cm long × 15 cm wide × 10 cm high [21]). Behind a perforated transparent plastic partition at one end of the arena and within a 4 × 15 cm backlit chamber, we placed, as a stimulus group, 80 first-instar nymphs belonging to the same population as the individual tested. Because individuals with a crowding history were not available at the time of the experiment, we used individuals with an isolation history (i.e., *MI *or *FI*), making our behavioural assay conservative. At the other end of the arena, there was a similar, but empty backlit chamber. The behaviour of the test insect was recorded on an event recorder in real time for 3 mn after introduction into the arena. We calculated, from the raw behavioural records for each test locust, values for eleven variables describing the attraction/repulsion of the insect to the backlit chambers of the arena and to the stimulus chamber separately, the tortuosity and speed of the locust track during the assay, some locomotory events, and the incidence of swaying (see Additional file 2 for a list of all eleven variables).

### Statistical analyses

Unless specified, statistical analyses were performed with the STATISTICA package v.6.1. Because morphometric and behavioural traits have been identified as key traits in *L. migratoria *phase change [25], we used them simultaneously in two multivariate analyses of morphometric and behavioural phases, respectively. To examine the potential interaction between population of origin (POPULATION) and rearing history (HISTORY) on *L. migratoria *morphometry and behaviour, we employed two full factorial MANOVAs on morphometric and behavioural data separately, with POPULATION and HISTORY as fixed factors. When appropriate, variables were Box-Cox transformed to conform to MANOVA assumptions, using JMP package v.3.2.2 (SAS Institute 1995, Cary, NC, USA; see Additional file 3 for details on Box-Cox transformations). We then used a canonical discriminant analysis to compare groups belonging to the same population of origin, i.e. Madagascar or France, but with histories of isolation and crowding through pairwise tests on Mahalanobis distances and plots of the canonical functions.

## Authors' contributions

The initial idea of the study was conceived by AE, ML, and YM. AE, MPC, and YM designed the experimental protocol. AF and MPC constructed the rearing racks and cages. ML provided the Malagasy insects. AF and MPC provided the French insects. AAS, AF, and MPC reared the locusts. AAS and MPC collected the morphometrical and behavioural measures. MPC carried out the statistical analyses and drafted the manuscript with substantial help from YM. AE gave valuable comments on the manuscript. All authors read and approved the final manuscript.

## Supplementary Material

Additional file 1 **Details of the canonical discriminant analyses of the measurements of four morphometric variables and eleven behavioural variables**. *CF1*, First canonical function; *CF2*, Second canonical function. Details of abbreviations for morphometrical and behavioural variables are provided in the Additional file 2. Click here for file

Additional file 2 **Illustration of the measurements used for calculating the four morphometric variables (from Dirsh 1953) (a) and list of the eleven behavioural variables (b)**. *E*, the length of the fore wing; *F*, the length of the hind femur; *C*, the maximum width of the head; *H*, the maximum height of the pronotum; *P*, the length of the pronotum; *V*, the minimum distance between the eyes. Click here for file

Additional file 3 **Box-Cox transformations for the four morphometric variables and the eleven behavioural variables**. The formulae presented in the table correspond to the transformations yielding the best fit to the normality hypothesis among the families of transformations $x=\frac{x^{\lambda }-1}{\lambda x^{\lambda -1}}$ if *λ *≠ 0, or, *x *= *x *ln(*x*) if *λ *= 0. Because raw behavioural data distributions included negative and null values, we added 2 at each raw behavioural value to conform to the requirement of strictly positive *x *values of the Box-Cox transformation families. Details of abbreviations for morphometrical and behavioural variables are provided in the Additional file 2. Click here for file
