# Supplementary material for: Genetic variation for parental effects on the propensity to gregarise in Locusta migratoria
Source: BMC Evol Biol. 2008 Feb 1;8:37. doi: 10.1186/1471-2148-8-37 (PMC2276201; doi:10.1186/1471-2148-8-37)
Supplement: Additional file 1 — Details of the canonical discriminant analyses of the measurements of four morphometric variables and eleven behavioural variables. CF1, First canonical function; CF2, Second canonical function. Details of abbreviations for morphometrical and behavioural variables are provided in the Additional file 2. [file 1471-2148-8-37-S1.PDF]

	Morphometry			Behaviour		
		CF1	*CF2*		*CF1*	*CF2*
Eigenvalues		1.020	0.401		0.606	0.084
Standardized coefficient for each variable	*E/F*	1.078	0.234	X_d	0.015	-0.107
	F/C	0.353	0.353	$\%_S$	0.029	0.108
	H/P	0.028	0.490	$\%_W$	-0.091	0.143
	V	-0.263	0.592	*St*	0.125	-0.032
				Sp	1.019	-0.768
				A	0.154	0.032
				T/t	-0.708	0.118
				W	-0.473	0.551
				J	-0.636	0.025
				C	0.616	0.427
				S	0.132	0.844
Group centroid values	MI	1.225	0.921	MI	1.298	0.217
	MC	0.782	-0.837	MC	0.350	-0.411
	FI	-1.228	0.200	FI	-0.658	-0.135
	FC	-0.531	-0.093	FC	-0.586	0.333
