# Supplementary material for: Genetic variation for parental effects on the propensity to gregarise in Locusta migratoria
Source: BMC Evol Biol. 2008 Feb 1;8:37. doi: 10.1186/1471-2148-8-37 (PMC2276201; doi:10.1186/1471-2148-8-37)
Supplement: Additional file 2 — Illustration of the measurements used for calculating the four morphometric variables (from Dirsh 1953) (a) and list of the eleven behavioural variables (b). E, the length of the fore wing; F, the length of the hind femur; C, the maximum width of the head; H, the maximum height of the pronotum; P, the length of the pronotum; V, the minimum distance between the eyes. [file 1471-2148-8-37-S2.PDF]

(a)

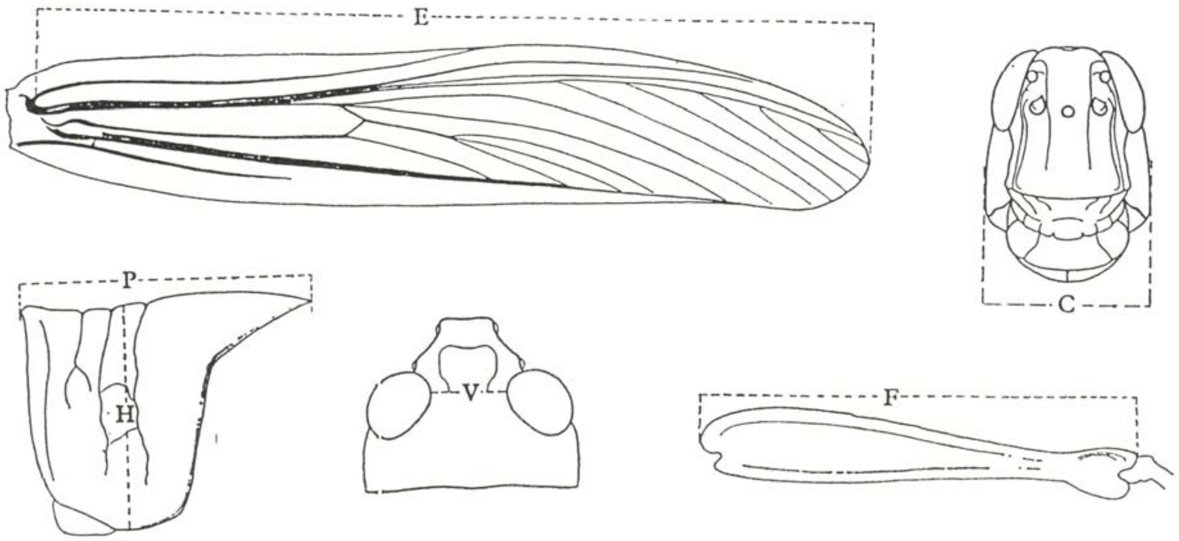

(b)

X_d	the final x-coordinate position of the insect in the arena
$\%_S$	the percentage of the time spent on the stimulus side of the arena (1/3 of total surface)
$\%_W$	the percentage of the time spent near the lighted walls of the arena (1/7 of total surface)
St	the track straightness
Sp	the track speed
A	the mean track angle
T/t	the turns per time
W	the walking time
J	the jumping frequency
C	the climbing time
S	the lateral or up and down swaying frequency of head and/or body with raised legs, but without leg movement
