# Supplementary material for: Genetic variation for parental effects on the propensity to gregarise in Locusta migratoria
Source: BMC Evol Biol. 2008 Feb 1;8:37. doi: 10.1186/1471-2148-8-37 (PMC2276201; doi:10.1186/1471-2148-8-37)
Supplement: Additional file 3 — Box-Cox transformations for the four morphometric variables and the eleven behavioural variables. The formulae presented in the table correspond to the transformations yielding the best fit to the normality hypothesis among the families of transformations x=xλ−1λxλ−1 if λ ≠ 0, or, x = x ln(x) if λ = 0. Because raw behavioural data distributions included negative and null values, we added 2 at each raw behavioural value to conform to the requirement of strictly positive x values of the Box-Cox transformation families. Details of abbreviations for morphometrical and behavioural variables are provided in the Additional file 2. [file 1471-2148-8-37-S3.PDF]

Morphometry		Behaviour	
E/F	$\frac{x^2 - 1}{3.511}$	X_d	$\frac{(x+2)^{-2} - 1}{-0.757}$
F/C	$\frac{x^{-2} - 1}{-0.045}$	$\%_S$	$\frac{(x+2)^{-2} - 1}{-0.173}$
H/P	$\frac{x^{-2} - 1}{-2.427}$	$\%_W$	$\frac{(x+2)^{1.2} - 1}{1.442}$
V	$\frac{x^{1.6} - 1}{2.971}$	*St*	$\frac{(x+2)^{-2} - 1}{-0.146}$
		Sp	$\frac{(x+2)^{-2} - 1}{-0.186}$
		A	$\frac{(x+2)^{0.8} - 1}{0.353}$
		T/t	$\frac{(x+2)^{-2} - 1}{-0.232}$
		W	$\frac{(x+2)^{-2} - 1}{-0.220}$
		J	$\frac{(x+2)^{-2} - 1}{-0.243}$
		C	$\frac{(x+2)^{-2} - 1}{-0.194}$
		S	$\frac{(x+2)^{-2} - 1}{-0.248}$
